# Risk factors associated with the intensity of COVID-19 outbreaks in Canadian community settings: a retrospective analysis of outbreak-level surveillance data

**DOI:** 10.1186/s12889-024-19853-4

**Published:** 2024-09-04

**Authors:** Demy Dam, Michelle Chen, Erin E. Rees, Bethany Cheng, Lynn Sukkarieh, Erin McGill, Yasmina Tehami, Anna Bellos, Jonathan Edwin, Kaitlin Patterson

**Affiliations:** 1 Centre for Emerging and Respiratory Infections and Pandemic Preparedness, Infectious Diseases and Vaccination Programs Branch, Public Health Agency of Canada, 130 Colonnade Rd, Ottawa, ON K1A 0K9 Canada; 2 Public Health Risk Sciences Division, National Microbiology Laboratory, Public Health Agency of Canada, 3200 rue Sicotte, Saint-Hyacinthe, QC J2S 2M2 Canada; 3 Groupe de recherche en épidémiologie des zoonoses et santé publique (GREZOSP), Faculty of Veterinary Medicine, Université de Montréal, Saint-Hyacinthe, QC Canada; 4 Department of Pathology and Microbiology, Faculty of Veterinary Medicine, Université de Montréal, Saint-Hyacinthe, QC Canada

**Keywords:** COVID-19, Community settings, Outbreak, Regression analysis

## Abstract

**Background:**

The severity of COVID-19 outbreaks is disproportionate across settings (e.g., long-term care facilities (LTCF), schools) across Canada. Few studies have examined factors associated with outbreak severity to inform prevention and response. Our study objective was to assess how outbreak severity, as measured using outbreak intensity and defined as number of outbreak-associated cases divided by outbreak duration, differed by setting and factors known to influence SARS-CoV-2 transmission.

**Methods:**

We described outbreak intensity trends in 2021 using data from the Canadian COVID-19 Outbreak Surveillance System from seven provinces/territories, representing 93% of the Canadian population. A negative binomial fixed-effects model was used to assess for associations between the outcome, outbreak intensity, and characteristics of outbreaks: setting type, median age of cases, number at risk, and vaccination coverage of at least 1 dose. Also included were variables previously reported to influence SARS-CoV-2 transmission: stringency of non-pharmaceutical interventions (NPI) and the predominant SARS-CoV-2 variant detected by surveillance.

**Results:**

The longest outbreaks occurred in LTCF (mean = 25.4 days) and correctional facilities (mean = 20.6 days) which also reported the largest outbreaks (mean = 29.6 cases per outbreak). Model results indicated that outbreak intensity was highest in correctional facilities. Relative to correctional facilities (referent), the second highest adjusted intensity ratio was in childcare centres (intensity ratio = 0.58 [95% CI: 0.51–0.66]), followed by LTCF (0.56 [95% CI: 0.51–0.66]). Schools had the lowest adjusted intensity ratio (0.46 [95% CI: 0.40–0.53]) despite having the highest proportion of outbreaks (37.5%). An increase in outbreak intensity was associated with increases in median age, the number at risk, and stringency of NPI. Greater vaccination coverage with at least 1 dose was associated with reduced outbreak intensity.

**Conclusion:**

Descriptive and multivariable model results indicated that in Canada during 2021, outbreak intensity was greatest in closed congregate living facilities: correctional facilities and LTCF. Findings from this study support the importance of vaccination in reducing outbreak intensity when vaccines are effective against infection with circulating variants, which is especially important for closed congregate living facilities where NPIs are more challenging to implement.

**Supplementary Information:**

The online version contains supplementary material available at 10.1186/s12889-024-19853-4.

## Background

Early in the COVID-19 pandemic, settings, such as long-term care facilities (LTCF), retirement homes, and correctional facilities experienced severe outbreaks, with high attack rates and mortality rates [1–5]. Conversely, outbreaks in schools and childcare settings had lower attack rates and fewer cases [6–8]. These observations highlighted the importance of monitoring infection trends and assessing for associated risk factors by setting.

In January 2021, the Public Health Agency of Canada (PHAC), in partnership with the Canadian provinces and territories (P/T), launched the Canadian COVID-19 Outbreak Surveillance System (CCOSS). Outbreak surveillance enhances case-based surveillance by capturing additional information such as the types of settings affected, outbreak duration and number of cases, and for some P/Ts, could link case-level data to outbreak data [9]. Surveillance data from outbreaks can be used for evidence-informed risk analyses aimed at mitigating these outbreaks. This is especially challenging in essential settings, such as correctional facilities and LTCF, where it is less feasible to implement certain public health measures (PHM) (e.g., closures, isolation, physical distancing) [10–12].

In the literature, different indicators have been used to characterise outbreak severity. Studies analyzing data from LTCF reported using measures such as proportion of facilities experiencing outbreaks, outbreak case counts, hospitalizations, deaths, and duration to characterize outbreak severity, while others used attack rates [13, 14]. Although the aforementioned indicators provide useful information on outbreaks, denominators on facilities and population at risk are often unavailable or not routinely collected, making it difficult to monitor outbreak severity across setting types and locations. Herein, we leveraged national surveillance data from CCOSS to assess risk factors associated with outbreak severity and propose the outbreak intensity indicator calculated as the number of outbreak cases divided by the outbreak duration in days. This indicator can be advantageous over attack rates (i.e. number of cases divided by the population at risk for a defined period of time) when the data for the population at risk are unavailable. During public health emergencies, resource limitations can restrict the types of surveillance data that are collected. Surveillance data for cases and outbreaks are better populated than data for the population at risk, as such, it is important to develop methods that best capitalize on the available data variables.

The objective of this study was to assess the risk factors associated with our proposed indicator for outbreak intensity within Canada and account for the COVID-19 context of transmission and public health measures in P/Ts over time. We discuss implications of the study results for prevention and response to infectious disease outbreaks.

## Methods

### Outbreak surveillance data

This analysis included outbreaks declared between January 1, 2021 and December 31, 2021. Of the P/Ts reporting to CCOSS, data for this study were available from: British Columbia, Alberta, Manitoba, Ontario, Quebec, Nova Scotia, and Prince Edward Island [9]. The P/Ts had variations in data elements submitted to CCOSS, but the common variables included: outbreak identifier (ID), number of outbreak cases, symptom onset dates of first and last outbreak cases, declaration dates for the start and end of the outbreak, type of setting, outbreak severity (number of hospitalized cases, number of deaths) and outbreak status (resolved or active). In 2021, CCOSS defined outbreaks as: “*Two or more confirmed cases of COVID-19 epidemiologically linked to a specific setting and/or location. Excluding households*,* since household cases may not be declared or managed as an outbreak if the risk of transmission is contained. This definition also excludes cases that are geographically clustered (e.g.*,* in a region*,* city*,* or town) but not epidemiologically linked*,* and cases attributed to community transmission* [15]*”.* Outbreaks were declared over at the discretion of the public health unit after a period of no new cases; most jurisdictions defined this period as two incubation periods with no new cases. Outbreaks were excluded from the analysis if they remained active by the end of the study period, had fewer than two cases, missing case counts, or had missing or nonsensical data on outbreak duration (e.g., negative, zero, one-day values).

For P/Ts that provided an outbreak ID in their case-level surveillance data that matched the outbreak ID submitted to CCOSS (Ontario, Quebec, Prince Edward Island), data for cases associated with the outbreaks were extracted from the PHAC National COVID-19 Case Dataset, a case-based surveillance system that collects data on demographics, clinical status, vaccination, and variant lineages of COVID-19 cases in Canada. The outbreak ID was used to perform linkage with case-level data, which were used to impute missing data and create outbreak-level variables for this analysis.

### Outcome variable

The study outcome is outbreak intensity: outbreak case counts divided by the outbreak duration in days. Outbreak duration represents the difference between the symptom onset dates of the first and last cases within an outbreak [16]. If the outbreak case count or duration were missing and linkage to case-level surveillance data was possible, then case-level data were used to obtain outbreak case counts and the onset dates of the first and last outbreak-associated cases. Outbreaks with missing onset dates of the last case (< 5%) were excluded from this analysis. If the onset dates of the first outbreak case were missing and case linkage was not possible, data imputation was used to populate the missing onset dates. For this, conditional multiple imputation was used with predictive mean matching, where one of five imputed datasets was selected at random, and the imputed datasets were not pooled [17]. The predictor variables used in the imputation included P/T, setting type, outbreak case count, difference between the onset date of the last case and outbreak start date, the average of the R_t_ during the 7 days before the onset date of the first case, and the difference between the onset date of the first case and outbreak start date.

### Explanatory variables for outbreak-level effects

Explanatory variables hypothesized to have an association with the outcome variable represented effects at the levels of the outbreak and the P/T. Specific to the outbreak, and central to our study, was assessing for the effect of the setting type on the outcome variable. CCOSS contained data on 24 different types of settings. To standardize setting types among the P/Ts analyzed in this study, some settings were combined into a single setting category. This was done by assuming that the risk of SARS-CoV-2 exposure and infection was similar in the setting populations, considering the demographics (e.g., age), behaviours (e.g., contact rates), feasibility and effectiveness of mitigation strategies, and physical facility characteristics (e.g., congregate living) for each setting category. This aggregation resulted in a total of eight settings: acute care, childcare, correctional facility, industrial/agricultural, LTCF, school, retirement residence/assisted living, and other congregate living facility (Table 1).

**Table 1:** Types and description of settings included in this study

Setting Type	Definition
Acute care	Hospital or similar setting where patients receive short-term treatment for an injury or severe episode of illness, an urgent medical condition, or during recovery from surgery. Aggregated: hospitals, emergency departments, urgent care, transitional care, convalescent care, and short-term inpatient rehabilitation centres.
Childcare	Institutions offering supervision and care of multiple infants or young children during the daytime (excludes day/overnight camps). Aggregated: daycares, childcare centres, and home childcare.
Correctional facility	Institutions where persons who are incarcerated are housed for short-term or long-term. Aggregated: provincial jails and prisons, penitentiaries, and youth correction centres.
Industrial/agricultural	Workplace settings where goods are manufactured or where food is cultivated or processed. Aggregated: agri-food processing facilities, factories, mines, wholesales, distribution centres, constructions, and transportations.
Long-term care facility	Facilities that provide living accommodations for people who require full-time supervised care, including professional health services, personal care, and other services (meals, laundry, cleaning). Aggregated: private and public LTCF.
School	Schools from kindergarten to grade 12 (excluding post-secondary, adult, other education). Aggregated: primary, middle, and secondary schools.
Retirement residence/ assisted living	Facilities that provide housing to retired adults, seniors, and individuals with disabilities. Facilities vary by size and types of services offered such as hospitality and personal care services. Aggregated: retirement residences, independent living, assisted/supportive living, and group homes.
Other congregate living	Facilities where people (most or all of whom are not related) live or stay overnight and use shared spaces (excluding LTCF, retirement residence, assisted/supportive living group homes). Aggregated: residential treatment centres, transition centres, shelters, and student dormitories.

The median age of the outbreak cases was also included as a proxy for vulnerability to SARS-CoV-2 infection in the setting population. Median age was computed by taking the median age of cases linked to the outbreak ID for P/Ts where linkage was possible. For the other P/Ts with missing data on median age, we imputed the median age using the conditional multiple imputation process with predictor variables: P/T, setting type, P/T population [18], date outbreak was declared, outbreak case count, and median age of cases (using outbreaks from P/Ts where linkage was possible).

We also included a variable for the number of people at risk in the setting population as a proxy for contact rates and transmission events. As with age data, not all P/Ts provided this information. We imputed this variable using the conditional multiple imputation process separately for each setting with the following predictor variables: P/T, P/T population [18], date outbreak was declared, outbreak case count, and number of people at risk in the setting (using outbreaks from one P/T which provided these data). Additional predictor variables were used for some settings: number of setting facilities in the P/T (acute care, childcare centres, correctional facilities, LTCF, retirement residences/assisted living, schools), number of outbreak cases hospitalized (other congregate living), number of outbreak deaths (other congregate living), and number of students in the P/T (schools) [19–24].

We used vaccination coverage at the P/T level to derive vaccination coverage in the outbreak population. Data from the Canadian COVID-19 Vaccination Coverage Surveillance System (CCVCSS) were incorporated into the model as a P/T-level variable [25]. The CCVCSS included the proportion of the P/T population that had received at least one dose of the COVID-19 vaccine by age group and week. CCVCSS values were incorporated based on the median age of outbreak cases (as a proxy for population at risk) and onset date of the earliest case with a 14-day lag to account for time to build immunity [26]. Before May 2021, age groups were less granular in CCVCSS. During this period, outbreaks in schools and childcare settings were assigned 0% coverage as this population was not yet eligible for vaccination, while outbreaks in other settings were assigned vaccination coverage values based on the corresponding age groups.

### Explanatory variables for provincial and territorial-level effects

To account for the epidemiological processes between the setting types and their P/T that may have affected the outcome variable, we assumed data available at the P/T level were representative. For instance, we included a measure based on the effective reproduction number (R_t_) calculated at the P/T level as a proxy for community transmission of SARS-CoV-2 experienced by the outbreak. R_t_ is the average number of secondary infections generated by a single case in a population where some individuals are immune and control measures may be in place; R_t_ < 1, R_t_ = 1, and R_t_ > 1 indicate decreasing, stable, and increasing transmission, respectively [27]. The methodology for computing R_t_ estimates has been previously described [28]. In our analysis, we used the average R_t_ of the 7 days prior to the onset date of the first outbreak case to account for the median incubation period.

The stringency index (SI), a measure for the stringency of PHMs implemented at the P/T level, was used to derive a variable accounting for PHMs in place at the time of the outbreak. The SI is a composite measure based on a combination of nine PHM indicators ranging from 0 to 100 with larger numbers representing more stringent measures [28]. Calculations of the SI were based on the Oxford method [29] and adapted for the Canadian context as described elsewhere [28]. In our analysis, SI was calculated by taking the average SI of the 7 days prior to the onset date of the first case for each outbreak to account for the median incubation period.

Finally, as SARS-CoV-2 variants differed in their transmissibility and severity [30, 31], we included a categorical variable to define the predominant variant circulating in the P/T over three periods: pre-Delta, Delta, and Omicron. Variant period cut-points were identified using case-level data based on the proportion of sequenced cases in each P/T over time. The Delta period began when more than 50% of the sequenced cases consisted of the Delta variant. The same logic was applied to determine the start of the Omicron period. The pre-Delta period was identified as the time before the Delta period. This method is similar to what was done by Klein et al. [32].

### Statistical modelling approach

**Fig. 1:**
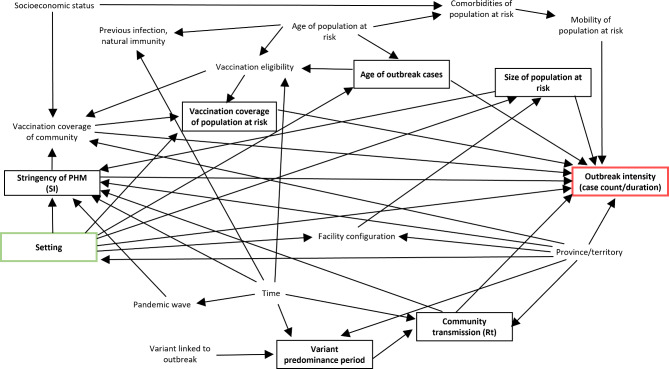
Directed acyclic graph of assumptions for factors associated with outbreak intensity. Legend: The exposure is represented in a green box, conditioned variables with observed data available for this study are represented in black boxes, and the outcome is represented in a red box. Variables not in boxes were not conditioned on. PHM: public health measures

We created a directed acyclic graph (DAG) to conceptualize theoretical associations between the study variables and help guide multivariable model building [33] (Fig. 1).

A negative binomial multivariable fixed-effects regression model was used to assess for the association of factors (e.g., setting, age of outbreak cases, vaccination coverage) with outbreak intensity using case count as the outcome and the logarithm of outbreak duration as the offset. We used a negative binomial regression modelling approach because the variance of the outcome exceeded the mean, indicating overdispersed data. Prior to model fitting, the continuous explanatory variables were standardized (mean = 0, standard deviation = 1) for a meaningful comparison of coefficients. We assessed P/T as a random effect to capture the variation that may have been caused by P/T-level differences in reporting and outbreak management. Explanatory variable selection was performed using stepwise forward selection (*p* < 0.05). Model selection was informed by the Akaike information criterion (AIC). Model residuals were graphically assessed to evaluate model fit and violations from parametric assumptions. Variable associations with the outcome, outbreak intensity, were quantified using intensity ratios. All statistical analyses were conducted in R [34].

The Pan-Canadian Public Health Network waived the need for approval and informed consent from originating parties as analyses were aggregated and conducted in accordance with the Multi-Lateral Information Sharing Agreement (MLISA) (see MLISA clause 20.d. and 20.f.i.A.). Ethics approval and consent to participate were not applicable as the study uses de-identified/anonymized, aggregated data drawn from existing surveillance systems that fall under the previously agreed upon MLISA between the Public Health Agency of Canada and the P/Ts.

## Results

A total of 19,005 outbreaks were included in this analysis. Descriptive results by setting type (Table 2) indicated that schools represented the highest proportion of reported outbreaks (37.5%), followed by industrial/agricultural (25.9%), and childcare settings (14.4%). Schools (29.4%) and industrial/agricultural settings (20.1%) also accounted for the highest proportions of outbreak-associated cases. Outbreaks in other congregate living settings were least intense (mean = 0.76 cases/day), followed by outbreaks in schools (mean = 0.77 cases/day), despite schools having the most outbreaks and the largest mean population at risk. In contrast, correctional facilities, the setting type reporting the fewest outbreaks, had the highest outbreak intensity (mean = 1.47 cases/day), the largest outbreaks (mean = 29.6 cases/outbreak), and the second longest duration (mean = 20.6 days). The longest outbreaks occurred in LTCF (mean = 25.4 days). Outlier outbreaks with short duration and high case counts, and long duration with low case counts, likely resulted from super-spreader events (i.e., high intensity) and sustained transmission (i.e., low intensity), respectively.

**Table 2:** Descriptive statistics of outbreak characteristics by setting type, January 1 to December 31, 2021

Setting	Outbreaks	Outbreak cases	Outbreak size (cases/ outbreak)	Outbreak duration (days)	Outbreak intensity (cases/day)	Number of people at risk (people)	Median age of outbreak cases (years)	Proportion of population at risk with at least 1 dose (%)
	Number (%)		Mean (95% CI)					
Acute care	842 (4.4)	8,945 (5.2)	10.6 (9.9,11.3)	15.2 (14.3, 16.1)	**0.91** **(0.85**,** 0.96)**	118 (98.8, 138.1)	58.8 (57.5, 60.0)	43.4 (40.5, 46.2)
Childcare	2,731 (14.4)	14,779 (8.6)	5.4 (5.2, 5.6)	9.0 (8.7, 9.3)	**0.84** **(0.81**,** 0.87)**	63 (60.5, 64.7)	10.6 (10.1, 11.0)	8.8 (7.8, 9.7)
Correctional facility	117 (0.6)	3,467 (2.0)	29.6 (22.6, 36.7)	20.6 (17.7, 23.5)	**1.47** **(1.19**,** 1.75)**	148 (129.5, 167.0)	36.4 (35.1, 37.7)	38.1 (31.2, 45.0)
Industrial/ agricultural	4,929 (25.9)	34,302 (20.1)	7.0 (6.6, 7.3)	10.6 (10.3, 11.0)	**0.81** **(0.79**,** 0.83)**	149 (141.9, 156.3)	41.3 (41.0, 41.6)	27.6 (26.7, 28.6)
Long-term care facility	1,120 (5.9)	30,584 (17.9)	27.3 (25.2, 29.4)	25.4 (24.0, 26.8)	**1.09** **(1.01**,** 1.17)**	234 (223.6, 244.5)	57.8 (56.8, 58.8)	54.8 (52.3, 57.2)
School	7,125 (37.5)	50,230 (29.4)	7.1 (6.8, 7.3)	12.7 (12.3, 13.0)	**0.77** **(0.76**,** 0.79)**	383 (375.4, 390.6)	12.2 (12.0, 12.4)	8.2 (7.7, 8.8)
Retirement residence/ assisted living	1,695 (8.9)	24,287 (14.2)	14.3 (13.4, 15.2)	19.0 (18.1, 19.8)	**0.93** **(0.87**,** 1.00)**	109 (104.5, 113.5)	58.6 (57.6, 59.6)	50.8 (48.9, 52.8)
Other congregate living	446 (2.3)	4,452 (2.6)	10.0 (8.8, 11.2)	16.7 (14.9, 18.4)	**0.76** **(0.70**,** 0.83)**	87 (79.4, 93.8)	39.6 (38.3, 41.0)	40.9 (37.2, 44.7)
Overall	19,005	171,046	9.0 (8.8, 9.2)	13.2 (13.0, 13.4)	**0.84** **(0.82**,** 0.85)**	223 (218.9, 227.0)	29.2 (28.9, 29.5)	22.4 (21.9, 22.9)

Legend: Bolded text highlights the outcome

**Fig. 2:**
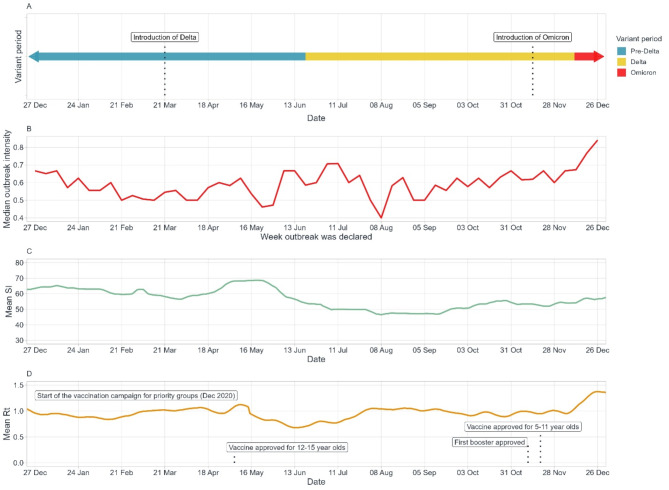
Temporal evolution of study variables from January to December 2021 for the seven assessed provinces/territories. Legend: (**A**) SARS-CoV-2 variant periods and introduction dates of the Delta and Omicron variants, (**B**) weekly median outbreak intensity, unweighted mean of the (**C**) stringency index (SI), and (**D**) effective reproduction number (R_t_) and vaccine rollout timelines. Date labels represent week start

After the vaccine rollout began in December 2020, there was a decrease in R_t_ followed by a slower decrease in the overall outbreak intensity at the national level (Fig. 2). Stringency of PHM was then eased from January to April 2021. Increases in R_t_ were observed as new variants of concern (VOCs) were detected in Canada (e.g., Delta introduction in March 2021, Delta-driven resurgence in cases in July 2021), while increases in the SI generally lagged behind R_t_ increases. Outbreaks with the highest intensity were in December 2021, corresponding with the introduction of the more transmissible and immuno-evasive Omicron variant [30, 31]. Outbreak intensity trends generally aligned with R_t_ trends over time, while SI and R_t_ generally had an inverse relationship given the lag in the increase and relaxation in SI following new VOCs and stabilization of cases, respectively.

### Model results

The best model was a fixed-effects negative binomial regression by lowest AIC (Table 3). The P/T random effect was not retained as it did not account for variation and did not improve model fit (higher AIC). All setting types had significantly lower outbreak intensity compared to correctional facilities. Schools had the lowest intensity (intensity ratio 0.46; 95% CI: 0.40–0.53), while childcare centres had the highest intensity (intensity ratio 0.58; 95% CI: 0.51–0.66) compared to correctional facilities. An increase in vaccination coverage with at least 1 dose was associated with reduced outbreak intensity. Holding other factors constant, outbreak intensity increased when there were increases in SI, number at risk, and higher median age of cases. Compared to the pre-Delta period, outbreak intensity was 1.43 and 2.17 times higher in the Delta and Omicron periods, respectively. R_t_ was not significantly associated with outbreak intensity.

**Table 3:** Negative binomial fixed-effects results for the adjusted intensity ratio estimates, 95% confidence intervals (CI), and the p-value

Variable	Adjusted intensity ratio (95% CI)	*p*-value
Setting		
Correctional facility	ref.	ref.
Acute care	0.51 (0.44–0.59)	< 0.0001
Childcare	0.58 (0.51–0.66)	< 0.0001
Industrial/agricultural	0.52 (0.45–0.59)	< 0.0001
Long-term care facility	0.56 (0.48–0.64)	< 0.0001
School	0.46 (0.40–0.53)	< 0.0001
Retirement residence/assisted living	0.50 (0.44–0.57)	< 0.0001
Other congregate living	0.50 (0.43–0.58)	< 0.0001
Variant period		
Pre-Delta	ref.	ref.
Delta	1.43 (1.38–1.49)	< 0.0001
Omicron	2.17 (2.05–2.30)	< 0.0001
Stringency index	1.13 (1.11–1.14)	< 0.0001
Vaccination coverage	0.88 (0.87–0.90)	< 0.0001
Number at risk	1.13 (1.12–1.14)	< 0.0001
Median age	1.13 (1.10–1.15)	< 0.0001

## Discussion

Descriptive analyses from our study of 2021 CCOSS data by setting type indicated that outbreak case counts and outbreak intensity were highest in correctional facilities and outbreaks lasted the longest in LTCF. Even when accounting for risk factors known to affect SARS-CoV-2 transmission, fixed-effects negative binomial model results indicated that correctional facilities had the most intense COVID-19 outbreaks, while schools had the lowest outbreak intensity (intensity ratio = 0.46). Higher vaccination coverage had a protective effect on the intensity of outbreaks, while increases in stringency of PHM, size of population at risk, and median age of cases were positively associated with intensity.

Congregate living settings, such as LTCF, correctional facilities, and retirement residences, have been identified as high-risk settings for outbreaks and transmission of respiratory pathogens [11, 12]. In Canada, these settings were disproportionately affected by severe COVID-19 outbreaks (e.g., high attack rates, long sustained transmission) [1–5, 34]. Once outbreaks have begun, large resident capacity, difficulty isolating cases, number of services offered, ethnic concentration, PHM, and contact patterns between staff and residents can contribute to onward transmission and outbreak intensity [3, 11]. Correctional facilities are particularly vulnerable to intense outbreaks due to overcrowding, limited testing, high turnover, lower health profile of individuals, and lower vaccination uptake [2, 11, 35, 36]. Infrastructure challenges in older correctional facilities such as open-barred cells may also contribute to the spread of respiratory viruses [4].

Compared to retirement residences/assisted living and other congregate living (e.g., shelter, dormitory), LTCF have populations with more complex health needs. Understaffed facilities, staff rotation between facilities, low adherence to infection control policies, limited paid sick leave, low staff-to-resident ratio, multibed rooms, and poor ventilation in older buildings are challenges that may have further exacerbated the intensity of outbreaks in LTCF [12, 14, 37–39]. Additionally, our model showed that higher median age was associated with increased intensity of outbreaks; older populations in congregate living settings often experience heightened contact patterns with staff and other residents due to reduced mobility [1, 2]. Age is not the only risk factor, both the presence and number of comorbidities increase the risk of COVID-19 transmission [40, 41]. In late 2020, the National Advisory Committee on Immunization announced that the COVID-19 vaccine rollout in Canada be prioritized to residents and staff of LTCF among other high-risk settings [42]. As vaccination coverage increased in LTCF in 2021, there was a notable decline in the incidence of outbreaks in this setting type [7].

Higher uptake of at least 1 dose of a COVID-19 vaccine was found to be negatively associated with outbreak intensity, consistent with findings that partial immunization was still effective against symptomatic infection, particularly for pre-Omicron variants [26, 43]. Although not assessed in this study, high vaccination coverage for complete primary series likely contributed to the decrease in outbreak intensity. In fact, the proportion of people who were eligible for vaccination and had completed their primary vaccination series was 70% in August 2021 and rose to 80% in December 2021 [25]. Vaccines have been found to have a positive impact in mitigating outbreaks, reducing outbreak frequency and duration in care homes [44]. The immuno-evasive characteristics of the Omicron variant may have contributed to its increased transmissibility and could explain why following its introduction in Canada in late 2021, there was an increase in outbreak intensity across all settings [31].

Our regression model results indicated that childcare centres had the highest outbreak intensity when compared to correctional facilities. However, in contrast to other setting types, childcare centres were found to have lower attack rates and were less severe (e.g., smaller case counts, shorter duration, lower case fatality) [6–8]. Unlike schools, childcare cannot be done online and mostly remained open during the pandemic [45]. Children aged under 5 years in childcare settings were not eligible for vaccination in 2021 and were vulnerable to infection as PHM such as masking and physical distancing were not feasible in this population [6].

Schools reported the highest number of outbreaks in 2021 but had the least intense outbreaks, consistent with findings from other studies [46, 47]. This could be attributed to the fact that many PHM were in place in schools throughout 2021, particularly school closures and remote/hybrid learning, as well as masking, cohorting of students and teachers, and rigorous screening/testing practices [46]. Furthermore, following the rollout of the COVID-19 vaccine, there was a notable decrease in the incidence of school outbreaks in vaccine-eligible populations (e.g., secondary schools) [7].

Stringency of PHM often increased following case resurgences and introduction of new VOCs and increases in community transmission, R_t_ [28]. It is likely due to this lag that our model showed that increases in the stringency of PHM were positively associated with outbreak intensity which indicates that increases in SI were in response to a growing number of cases. The impact of increases in SI takes time to have an effect on community transmission which could further contribute to the positive correlation of SI with outbreak intensity [28]. Similarly, there was a delay in the lifting of PHM which may have also contributed to the observed counterintuitive relationship between stringency and outbreak intensity. Our model also found that size of the population at risk was associated with increased outbreak intensity. Outbreaks are more likely to be large and more intense if there is a larger population susceptible to infection and increased contact of the population within a setting [3]. PHM, such as cohorting, staggering shifts/timetables, and depopulation/discharge of patients and those incarcerated, have been used to reduce population sizes in various settings [1, 11, 48].

### Limitations

Data for this study were collected by P/Ts for surveillance purposes and P/Ts established links between outbreaks and cases. Although the national outbreak definition is applied to CCOSS outbreaks in this analysis, variations in outbreak definitions and testing policies by P/Ts and over time may not be completely accounted for. The definitions for declaring outbreaks varied across jurisdictions and over time with the emergence of new variants, but the potential bias that this would have introduced would have been minimal because jurisdictions used two incubation periods without new cases to declare an outbreak over. Case ascertainment may have been more likely in high-risk settings such as correctional facilities and LTCF compared to settings like schools. Case-level data (e.g., comorbidities) which could have further contributed to our understanding of risk factors associated with COVID-19 outbreaks were not available and could not be assessed. There may be bias towards lower outbreak intensity in more open settings (e.g., schools) as outbreaks in these settings may have been more likely to have multiple introductions, resulting in longer durations, compared to closed or semi-closed settings (e.g., prisons). Population at risk was not available for all outbreaks and had to be imputed based on data from one P/T. Additionally, the temporal distribution of cases within an outbreak was not available, nor were information for transmission dynamics (e.g., multiple introductions, resident-resident infection events, or staff-resident infection events) available. SI was included as a composite measure which precluded the ability to assess effects of individual PHM; the SI also does not account for public compliance [28]. The model only accounted for the proportion of the population having received at least 1 dose of a COVID-19 vaccine. However, throughout the second half of the study period, the proportion of those who completed a primary vaccination series (2 doses) was high, which may have affected outbreak intensity. Due to variations in the rollout of the second dose, vaccination coverage estimates for the completion of a primary series were not stable enough to include in the model. In addition, vaccination coverage estimates do not account for more granular geographical and setting/facility-level variations (e.g., LTCF had higher vaccination coverage), and the median age of outbreak cases had to be used as a proxy to estimate the vaccination coverage of the population at risk (i.e., within a facility) as it was not available. Although CCOSS data represent 93% of the Canadian population, certain populations may be underrepresented as data that would identify Indigenous settings or populations were not collected [9].

## Conclusion

Here, we have noted that outbreaks are most intense in closed congregate living facilities (e.g., correctional facilities, LTCF) and least intense in open settings where cases can be isolated, and the setting can be closed (e.g., schools). For SARS-CoV-2, this aligns with the mode of respiratory and aerosol transmission. In addition, significant association has been demonstrated between outbreak intensity and epidemiologically relevant contextual indicators such as vaccination coverage, stringency of PHM, population at risk, and median age.

The outbreak intensity indicator for outbreak severity could provide useful characterization of outbreaks between locations of a similar setting type or across settings. Population denominators and case exposure information are often unavailable, making attack rates and risk estimates difficult to calculate using surveillance data. This analysis demonstrates the resource-efficient use of population-level outbreak surveillance data for hypothesis generation to better understand risk and transmission dynamics of pathogens of epidemic and pandemic potential. Consideration should be made for capturing data that allows the calculation of outbreak intensity and the analysis of factors associated with setting-specific characteristics (e.g., crowding, ventilation); population characteristics (e.g., health, vaccination status); and structural characteristic (e.g., policies, PHM) to promote understanding of clinical and epidemiological parameters for novel pathogens or emerging transmission contexts for existing pathogens.

In Canada, high intensity outbreaks in settings that provide essential services underscore the importance of surveillance and enhanced PHM to prevent SARS-CoV-2 transmission. Further study is warranted to explore the use of outbreak intensity to inform our understanding of different types of pathogens and routes of transmission.

## Electronic supplementary material

Below is the link to the electronic supplementary material.

Supplementary Material 1

## Data Availability

The datasets generated and/or analyzed during the current study are not publicly available due to data sharing agreements in place with provinces and territories but are available from the corresponding author on reasonable request.

## Abbreviations

LTCF: Long-Term Care Facilities
NPI: Non-Pharmaceutical Interventions
PHM: Public Health Measures
PHAC: Public Health Agency of Canada
CCOSS: Canadian COVID-19 Outbreak Surveillance System
P/T: Provinces and Territories
DAG: Directed Acyclic Graph
Rt: Effective Reproduction Number
SI: Stringency Index of NPI
CCVCSS: Canadian COVID-19 Vaccination Coverage Surveillance System
AIC: Akaike Information Criterion
MLISA: Multi-Lateral Information Sharing Agreement
CI: Confidence Interval
VOC: Variants of Concern
