# Supplementary material for: Risk factors associated with the intensity of COVID-19 outbreaks in Canadian community settings: a retrospective analysis of outbreak-level surveillance data
Source: BMC Public Health. 2024 Sep 4;24:2409. doi: 10.1186/s12889-024-19853-4 (PMC11375942; doi:10.1186/s12889-024-19853-4)
Supplement: Supplementary file 1 — Supplementary Material 1 [file 12889_2024_19853_MOESM1_ESM.pdf]

Risk factors associated with the intensity of COVID-19 outbreaks in Canadian community settings: A retrospective analysis of outbreak-level surveillance data

Supplementary Materials

Table S1. Proportion of missing data by variable prior to imputing data and excluding outbreaks

Variable	Proportion of missing data (%)	Details on what was done with the missing data
Setting	0	N/A
Status	<0.1	Outbreaks with missing status were excluded.
Number of cases	0	N/A
Number of cases hospitalized	4.5	Variable was only used in multiple imputation models.
Number of cases deceased	4.5	Variable was only used in multiple imputation models.
Number of people at risk	85.9	Number of people at risk was only available for outbreaks in one province/territory.
Median age of outbreak cases	20.4	Data was only available for three jurisdictions where outbreak-case linkage was possible.
Median age of the population at risk	0	N/A
Date outbreak was declared	0	N/A
Date outbreak was declared over	0.2	Variable was only used in multiple imputation models.
Onset date of the earliest case	14.4	We used multiple imputation to populate missing onset dates of the first case for one jurisdiction where case linkage was not possible.
Onset date of the latest case	4.5	Outbreaks with missing onset date of the latest case were excluded.
Duration based on the administrative dates	0.2	Variable was only used to filter out outbreaks with a negative duration.
Duration based on the onset dates	18.8	After using multiple imputation to populate missing onset dates of the first case for one jurisdiction where case linkage was not possible, the proportion of missing data became 0%.
Vaccination coverage with at least 1 dose for the population at risk	0	N/A
Outbreak intensity based on onset date	18.8	After using multiple imputation to populate missing onset dates of the first case for one jurisdiction where case linkage was not possible, the proportion of missing data became 0%.
Effective reproduction	14.4	After using multiple imputation to populate missing onset dates of the first case for one jurisdiction where

number		case linkage was not possible, the proportion of missing data became <0.1%.<br><br>Outbreaks from one province/territory which did not have data for Rt were excluded (<0.1% missing).
Stringency index	0	N/A
Variant period	0	N/A

P/T: Province/territory

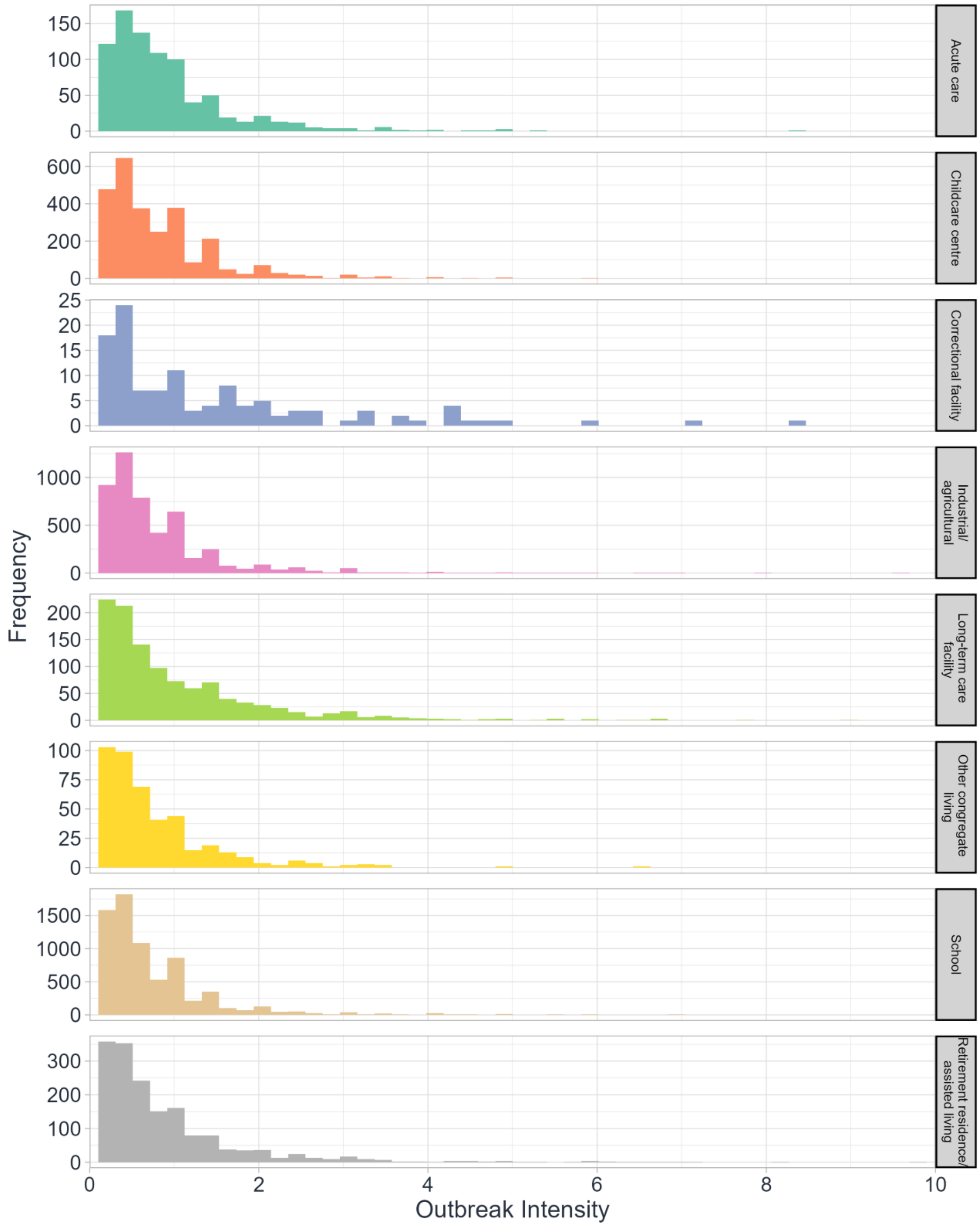

Figure S1. Distribution of outbreak intensity, by setting. Only a small proportion (<1%) of outbreaks in LTCF, schools, industrial/agricultural, and retirement residence/assisted living settings had intensities higher than 10 cases per day.

Table S2. Negative binomial fixed effects results, without correctional facilities, for the adjusted intensity ratio estimates, 95% confidence intervals (CIs), and p-values.

Variable	Intensity Ratio (95% CI)	p-value
Setting		
Childcare	ref.	ref.
Acute care	0.88 (0.82-0.95)	<.001
Industrial/agricultural	0.89 (0.84-0.93)	<.001
Long-term care facility	0.96 (0.89-1.03)	0.25
School	0.79 (0.76-0.83)	<.001
Retirement residence/assisted living	0.87 (0.81-0.93)	<.001
Other congregate living	0.87 (0.80-0.94)	<.001
VOC period		
Pre-Delta	ref.	ref.
Delta	1.43 (1.38-1.48)	<.001
Omicron	2.17 (2.04-2.29)	<.001
Stringency index	1.12 (1.11-1.14)	<.001
Vaccination coverage	0.88 (0.87-0.90)	<.001
Number at risk	1.13 (1.12-1.14)	<.001
Median age	1.13 (1.10-1.15)	<.001
